# Supplementary material for: scAbsolute: measuring single-cell ploidy and replication status
Source: Genome Biol. 2024 Mar 4;25:62. doi: 10.1186/s13059-024-03204-y (PMC10910719; doi:10.1186/s13059-024-03204-y)
Supplement: Supplementary file 1 — Additional file 1. Supplementary tables and figures. [file 13059_2024_3204_MOESM1_ESM.pdf]

Additional file 1: Supplementary tables and figures

Table S1: Overview over DLP+ sequencing data used in this study.

Sample	Library	number of cells
SA928	A73044A	958
SA928	A75616A	171
SA928	A75616B	183
SA928	A75616C	164
SA928	A75617A	209
SA928	A90553A	419
SA928	A90553C	1428
SA928	A90560A	228
SA928	A90648B	327
SA928	A90682	395
SA928	A90685	241
SA928	A90689A	175
SA928	A90689B	611
SA928	A90689C	596
SA928	A90694A	348
SA928	A90694B	908
SA928	A90706	272
SA1044	A96139A	1305
SA1047B	A96210B	639
SA1087	A96150A	590
SA1089	A96156B	344
SA1090	A96213A	709
SA1135	A96199A	460
SA922	A90554B	436

Table S2: cellenONE isolation parameters for diameter, elongation, circumference, and fluorescence values. Values in parenthesis indicate selection channel (positive/negative).

Sample	diameter (min,µm)	diameter (max,µm)	Elongation	Transmission	Blue channel	Green channel
PEO1-FUCCI (G1)	17	24	1.8	1-255 (+)	8-255 (+)	NA
PEO1-FUCCI (G2)	20	26	1.8	1-255 (+)	8-255 (+)	NA
PEO1-FUCCI (S)	19.3	23.9	1.76	1-255 (+)	8-255 (+)	NA
PEO1-PLOIDY	15.5	21	1.8	1-255 (+)	8-255 (+)	5-255 (-)

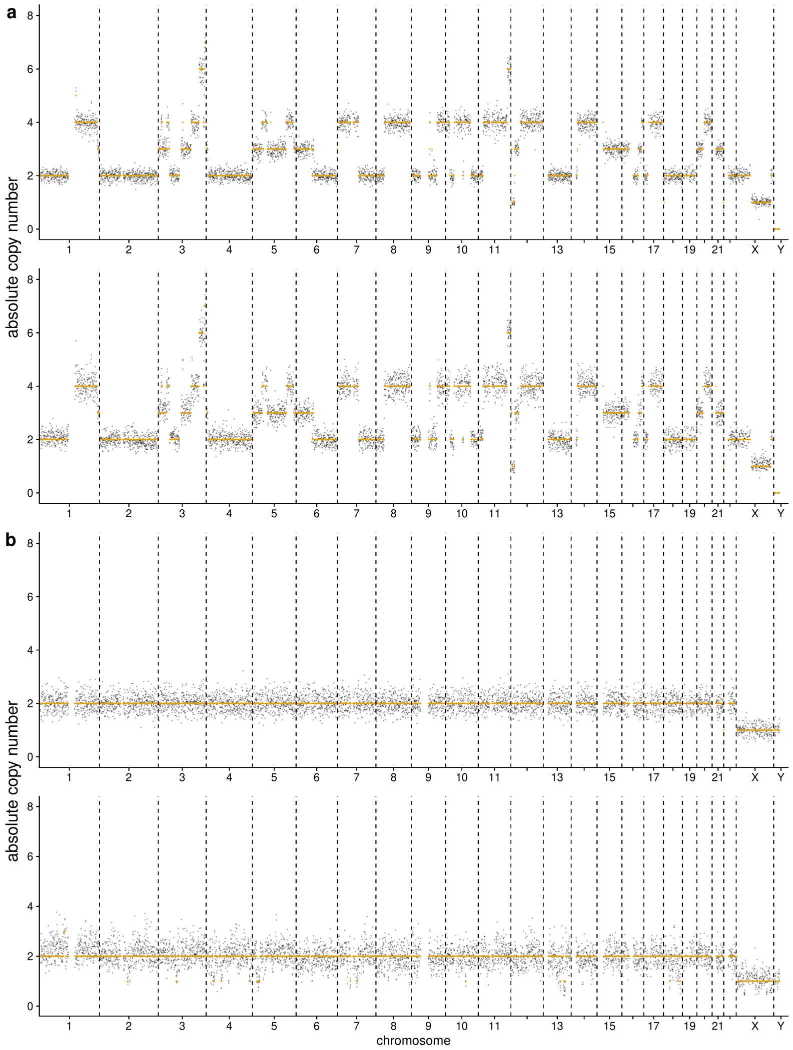

Fig. S1: **Example cells in G1 and G2 phase of cell cycle (a)** Example tumour cell (T-47D) in G1 phase (top panel) and G2 phase (bottom panel) of cell cycle. **(b)** Example normal cell (SA928) in G1 phase (top panel) and G2 phase (bottom panel) of cell cycle. In all cases, cell cycle stage has been verified by DAPI staining based FACS and by subsequent computational analysis. Note, that in case of G2 phase, the initial ploidy solution is off by a multiplicative factor of 2.

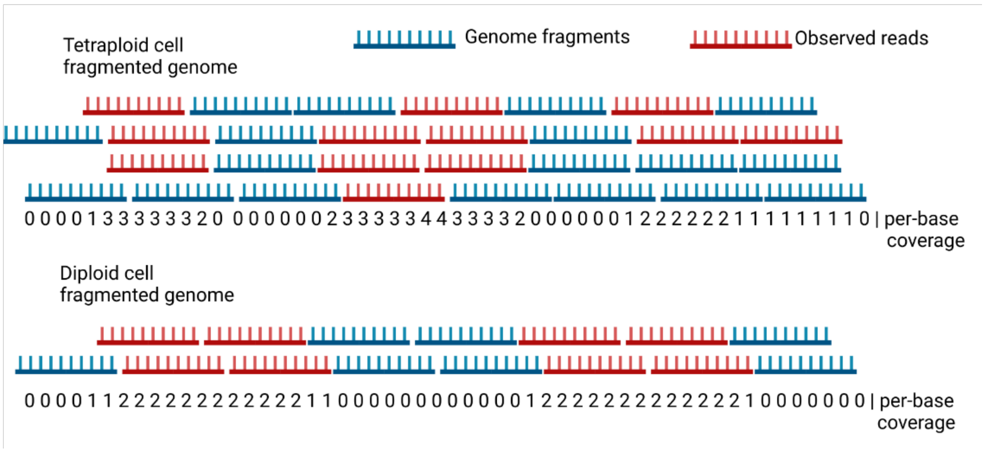

Fig. S2: **Schematic of read density measure.** Read density varies based on the underlying amount of DNA, and the number of overlapping reads can be used to infer the number of original DNA molecules, if the DNA has not been whole-genome amplified. If the DNA is not amplified, each read can be assigned to one strand of DNA in a cell. This makes the ploidy of a cell identifiable by the bound on the number of overlapping reads that can be observed per cell.

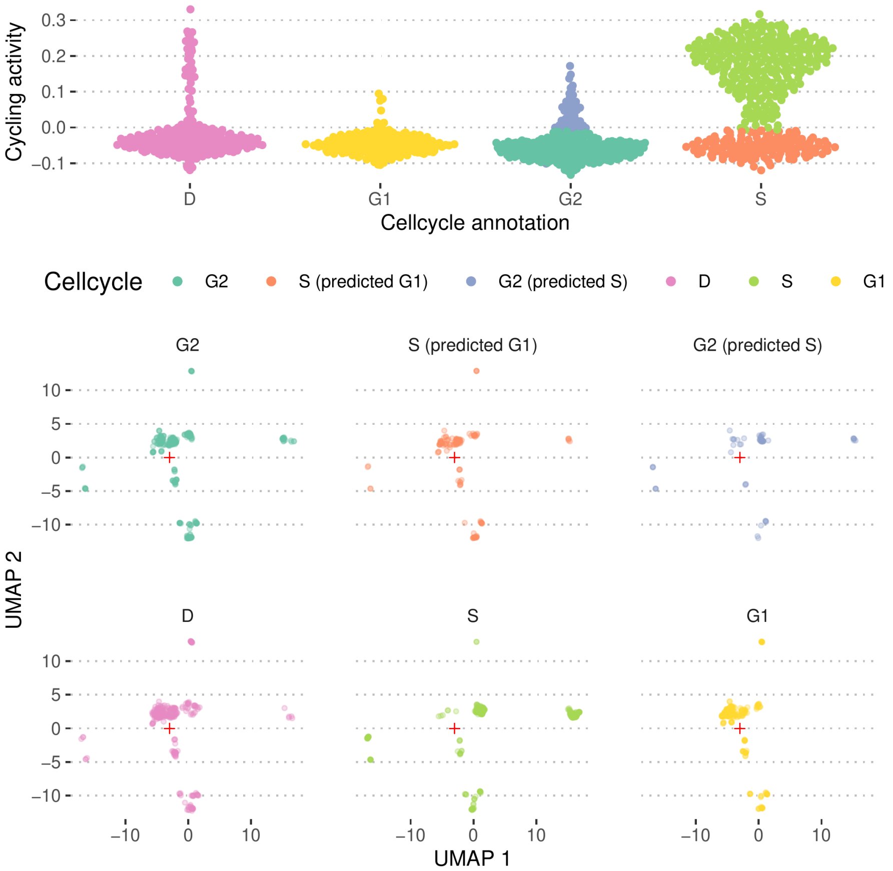

Fig. S3: ***scAbsolute* predictions for T-47D sample.** Top panel shows cycling activity predictions for cells from the T-47D cell line, with DAPI staining based FACS cell cycle annotation on the x-axis. Both for the S phase, and for the G2 phase annotated cells, we observe subgroups that are classified differently by the cycling activity predictor. Looking at copy number profiles in a UMAP representation, we can see that the groups cluster differently based on the cycling activity predictions. Cells that are annotated to be in S phase, but predicted to be in G1 phase (orange), appear to cluster closer with the G1 cells. Similarly, cells in G2 phase that have been predicted to be undergoing replication are clustering more similarly to the cells in S phase. The same pattern can be observed in the raw copy number profiles in Fig. S4.

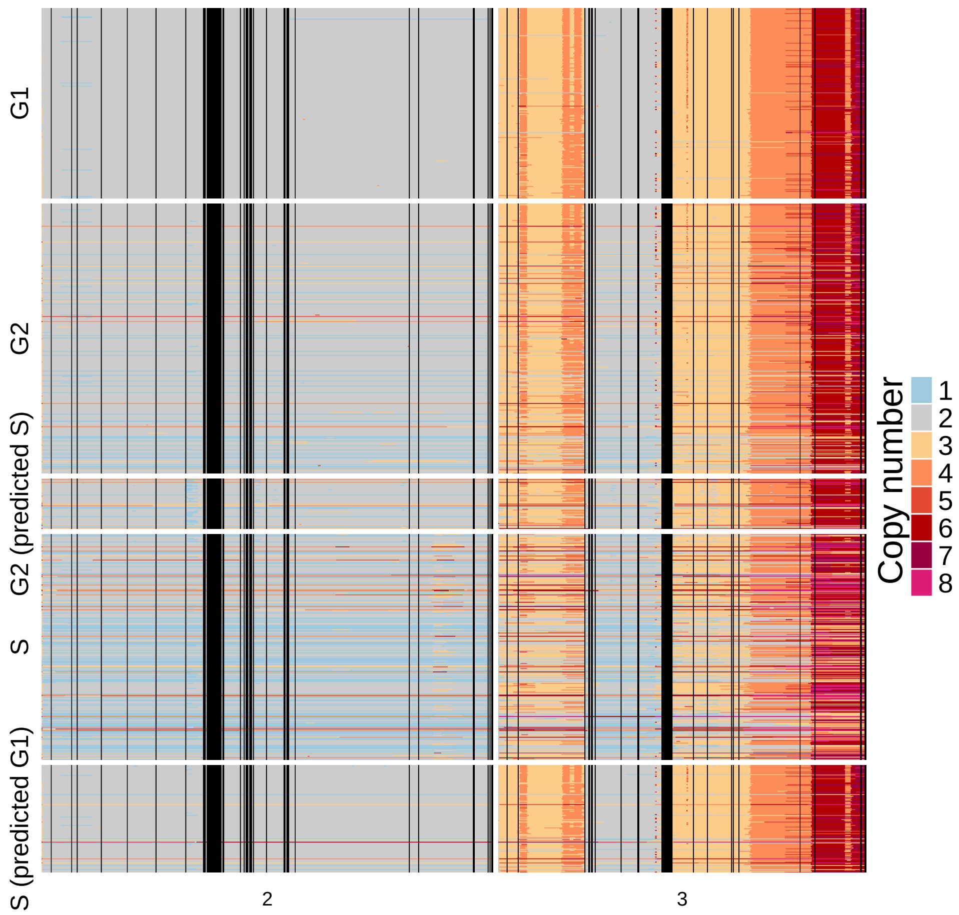

Fig. S4: **Initial copy number profiles as predicted by scAbsolute for DLP+ T-47D sample for chromosomes 2 and 3.** Overall, the vast majority of cells is independently called with the same ploidy. We see that there is a small subset of cells with the wrong ploidy solution and an even smaller group of cells that is otherwise classified wrongly. The algorithm cannot distinguish between G1/G2 cells. We observe some noise, characteristic of S phase cells that are also mentioned in the original publication among the G2 cell population. One can observe a clear difference in copy number profiles between S phase cells predicted to be in G1 phase of the cell cycle, and S phase cells that are predicted to be in S phase based on cycling activity. This might indicate an issue with the underlying ground truth data.

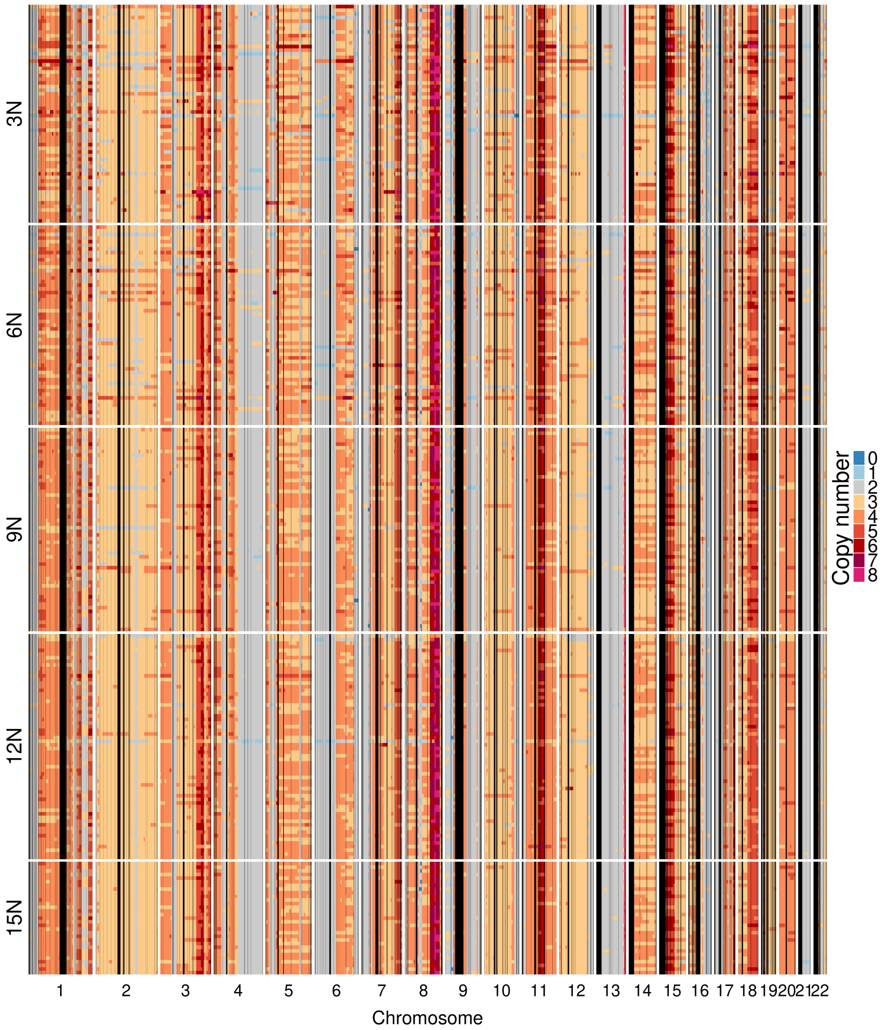

Fig. S5: **Copy number profiles for PEO1 cell multiplet experiment.** Combinations of multiple cells (1-5 cells) are artificially added to the same well and sequenced as a single cell. Here, we enforce a ploidy solution corresponding to the 3N normal state when fitting the copy number profiles. Visual inspection doesn't allow to distinguish the different ploidy solutions.

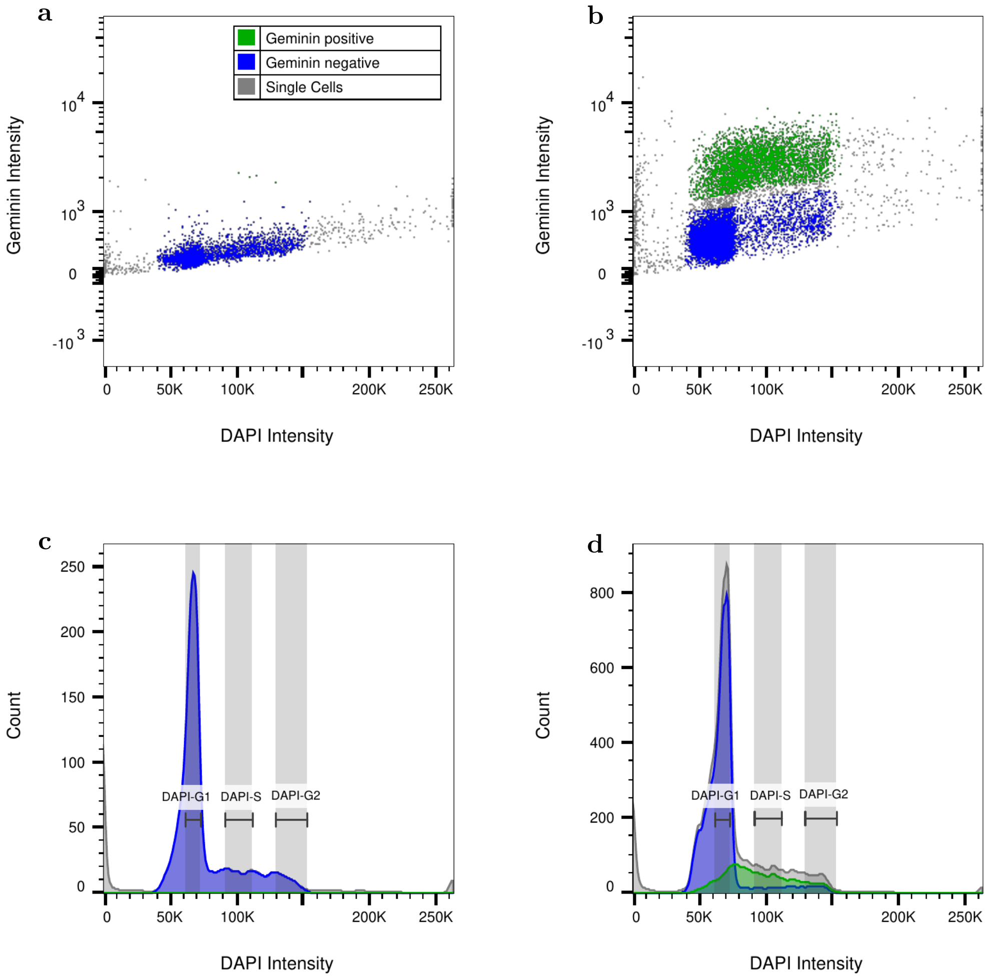

Fig. S6: **Flow cytometry images of normal cells (NA12878) stained with Geminin-AF488 and DAPI for improved G1 cell cycle sorting.** Geminin-positive populations — green; Geminin-negative populations — blue. **a)** Geminin-negative control – NA12878 stained with DAPI and AF488 secondary antibody. **b)** NA12878 stained with Geminin/AF488 and DAPI. Manual gating of Geminin-positive and Geminin-negative populations. **c)** Cell cycle curve of Geminin-negative control using DAPI intensity, with overlay of Geminin gating. DAPI-G1, DAPI-S and DAPI-G2 gating represents original flow cytometry sorting gates using DAPI alone for cell cycle analysis. **d)** Cell cycle curve of Geminin stained NA12878 using DAPI intensity. Overlay of Geminin gating reveals Early S phase cells leaking into G1 sorting using DAPI only.

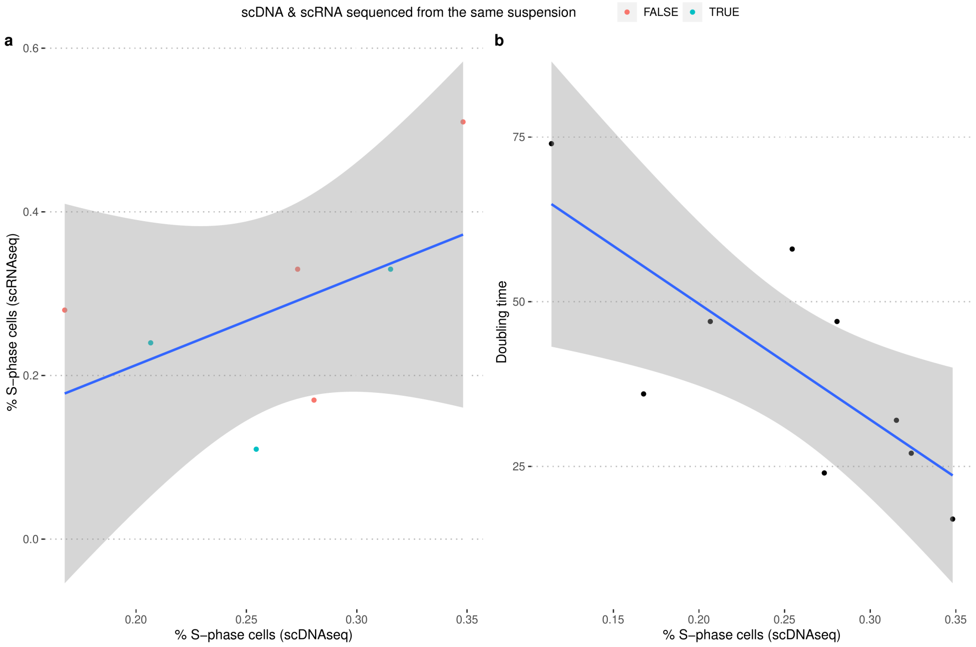

Fig. S7: **Validation measures for scDNAseq estimates of number of cycling cells in gastric cancer cell lines. (a)** The number of cycling cells as estimated in scDNAseq data corresponds to estimates of cycling cells based on scRNAseq data. **(b)** Doubling time of cell lines correlates negatively with number of cycling cells as estimated in scDNAseq data.

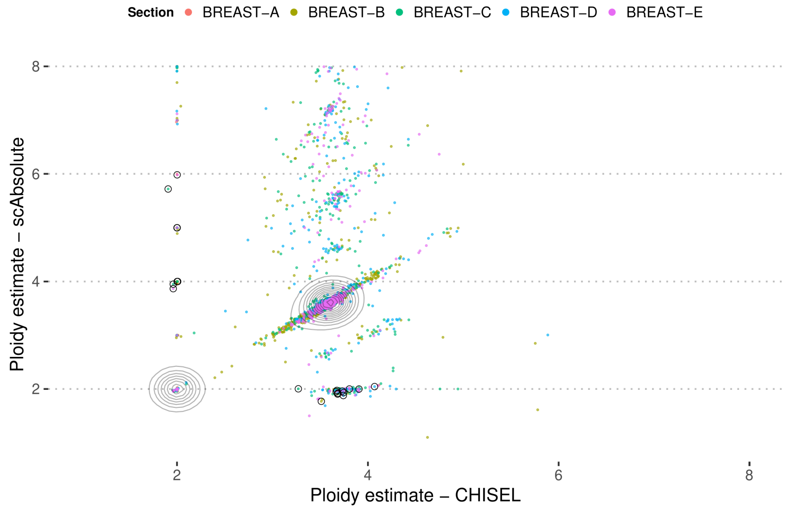

Fig. S8: **Ploidy predictions for *scAbsolute* and CHISEL on 10X Breast tumour sample.** Example cells, shown in Figs. S9 and S10 are marked with black circles. Density areas are indicated on the diagonal, showing a relatively large overlap of predictions in this particular tumour sample.

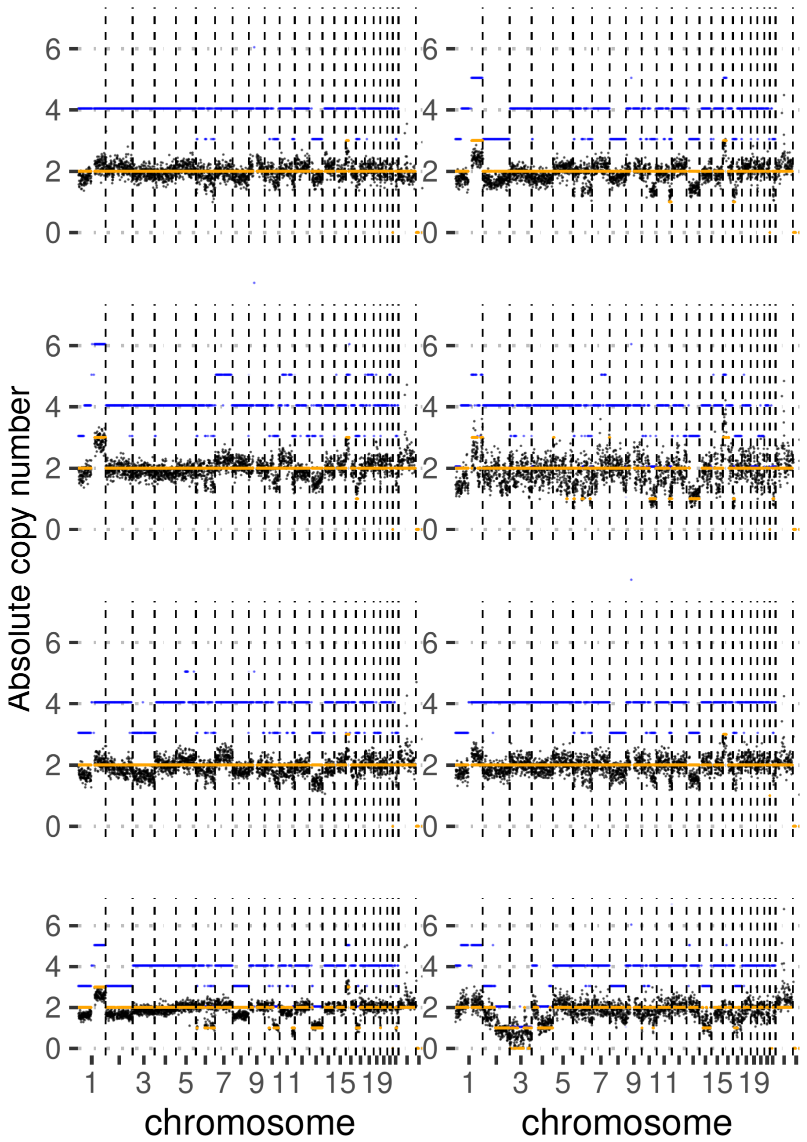

Fig. S9: **Randomly selected copy number profiles, for which *scAbsolute* predicts a diploid copy number profile and CHISEL has a contradictory prediction.** *scAbsolute* predictions are shown in orange, and CHISEL in blue. In some cases it appears that *scAbsolute* fails to detect slight copy number changes, indicating a more conservative segmentation. CHISEL tends to select higher ploidy solutions that might not necessarily be the minimum ploidy fit in these cases, as shown in the bottom row.

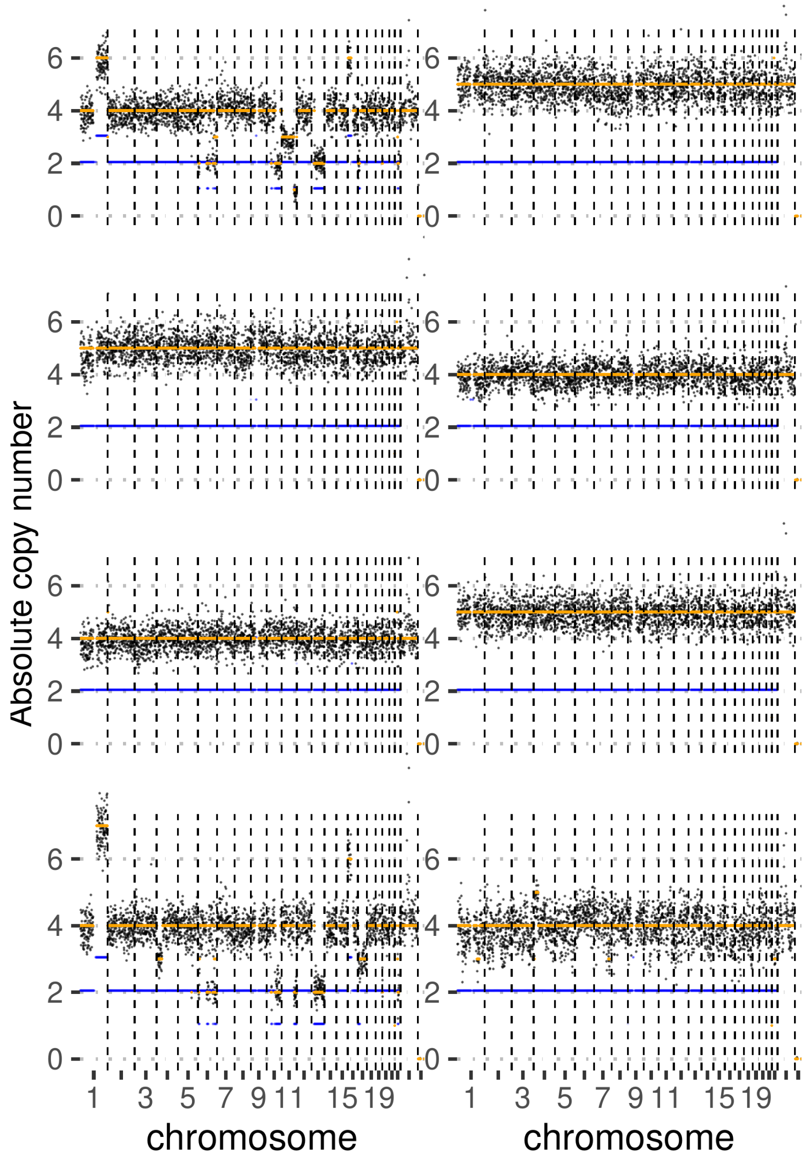

Fig. S10: **Randomly selected copy number profiles, for which CHISEL predicts a diploid copy number profile and *scAbsolute* has a contradictory prediction.** *scAbsolute* predictions are shown in orange, and CHISEL in blue. Here, *scAbsolute* selects potentially wrong solutions in cases with very minor erroneous copy number changes. Note that the number of these cases is very small (about 1% of cells). At the same time, it appears more accurate in cases with multiple levels of copy number changes (top left and bottom left examples).

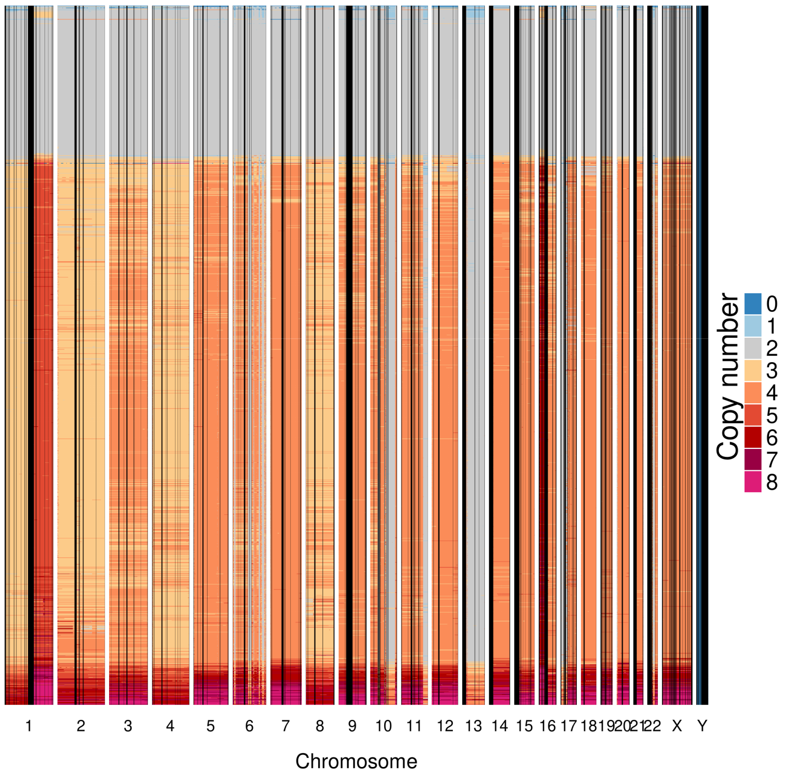

Fig. S11: **Copy number prediction for Patient S0 (section E) using *scAbsolute***. The prediction reflects the general copy number landscape for the sample as presented in Zaccaria and Raphael [58]. Note that the cells have not been quality controlled, and this explains the small number of ploidy outliers at high and low ploidies. Overall it appears relatively easy to detect higher ploidy states in this dataset, given the number of copy number segments at varying ploidy levels. This might be indicative of a relatively early WGD event leading to the observed copy number profiles.

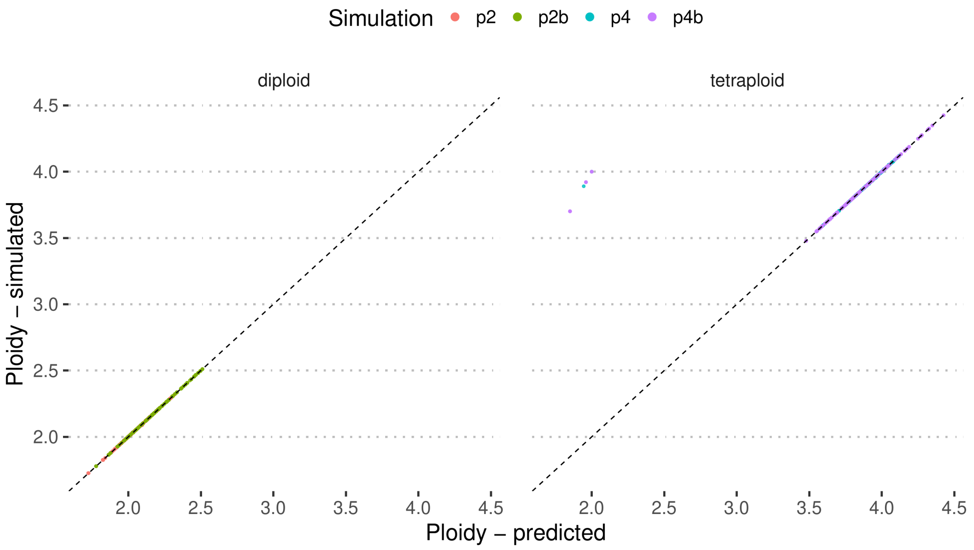

Fig. S12: **Performance on simulated diploid and tetraploid copy number profiles.** Evaluating ploidy predictions by *scAbsolute* on synthetic copy number profiles created with SCICoNE [64] copy number simulator. We have created copy number profiles with a base ploidy of 2 (p2, p2b), and 4 (p4, p4b). Predictions align well with groundtruth ploidy, with the exception of a small set of cells predicted as diploid for the p4 and p4b data sets (about 2% of cells are wrongly predicted to be diploid across the two simulations).

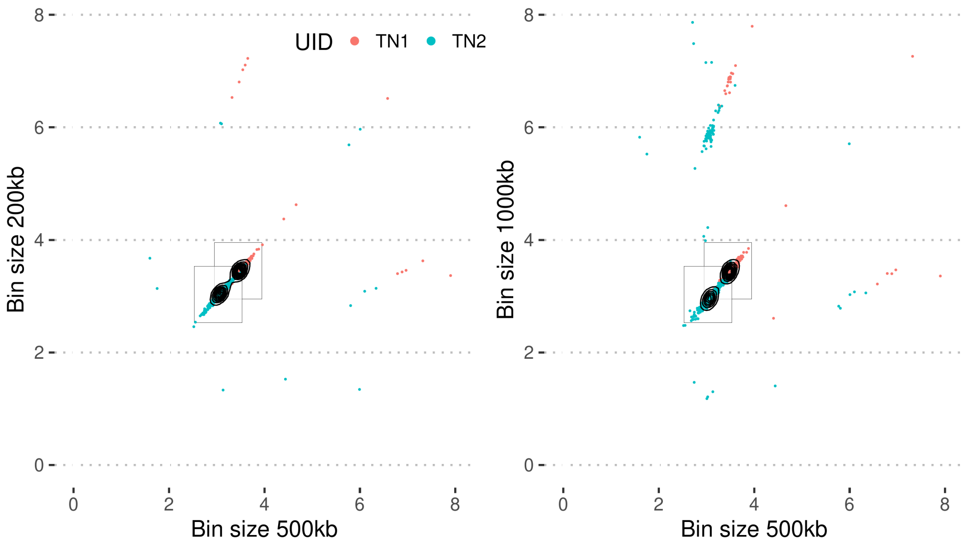

Fig. S13: **Congruence of ploidy predictions for varying bin sizes.** Ploidy predictions for two example data sets with relatively deep sequencing depth (TN1, TN2) for bin sizes of 200, 500, and 1000 kb. The point estimate (± 0.5) of FACS-based ploidy prediction is marked by black rectanges. Ploidy predictions mostly agree between the different bin sizes, with a slight increase in wrong ploidy predictions for 1000 kb.

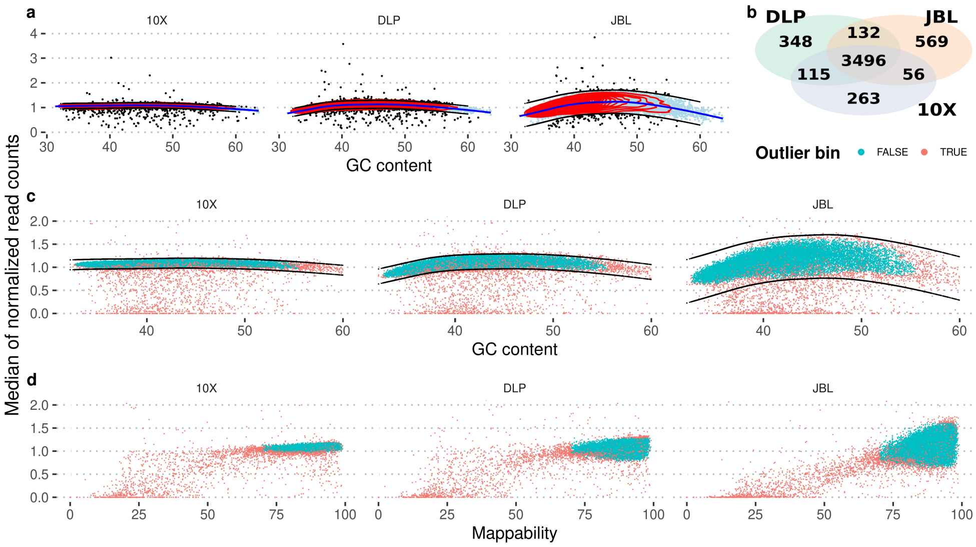

Fig. S14: **Bin level quality control for autosomes across sequencing technologies. a)** Generative additive model smoothing (blue line) and kernel density estimation (red contours) to identify genomic bins that have below or above average median expected read counts. **b)** Number of genomic bins identified as outliers by sequencing technology. We remove the union of all outliers. **c+d)** Genomic bins identified as outliers (in red) across different sequencing technologies as a function of GC content **(c)**, and mappability **(d)**.

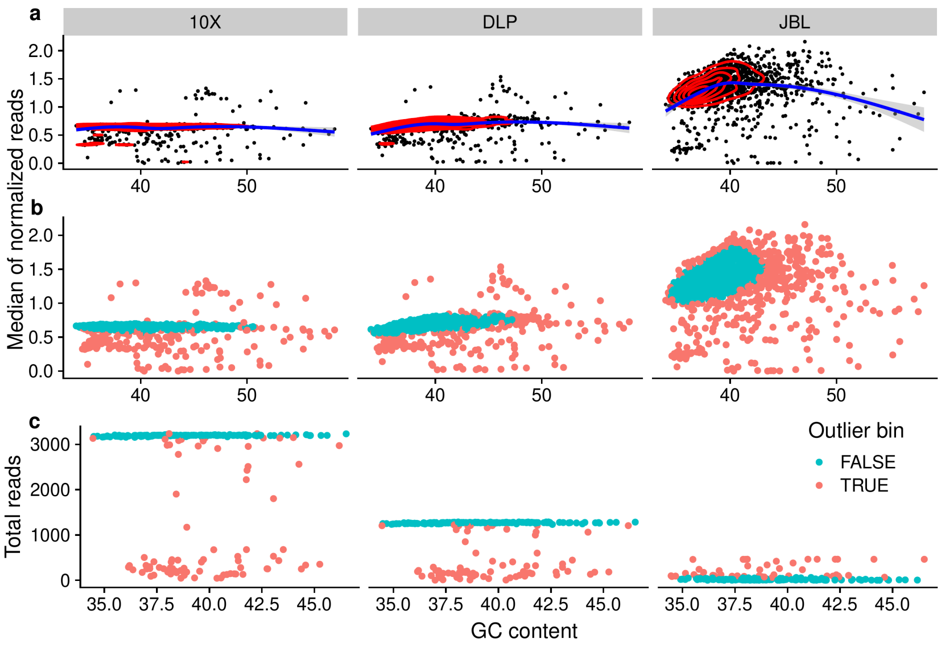

Fig. S15: **Bin level quality control for sex chromosomes across sequencing technologies. a)** Generative additive model smoothing (blue line) and kernel density estimation (red contours) to identify genomic bins that have below or above average median expected read counts for the X chromosome. **b)** Outlier bins that are removed based on the fits in a) are marked. **c)** Total (absolute) reads per bin observed on the Y chromosome. Outlier bins (based on deviation from median number of total reads) are marked in red.

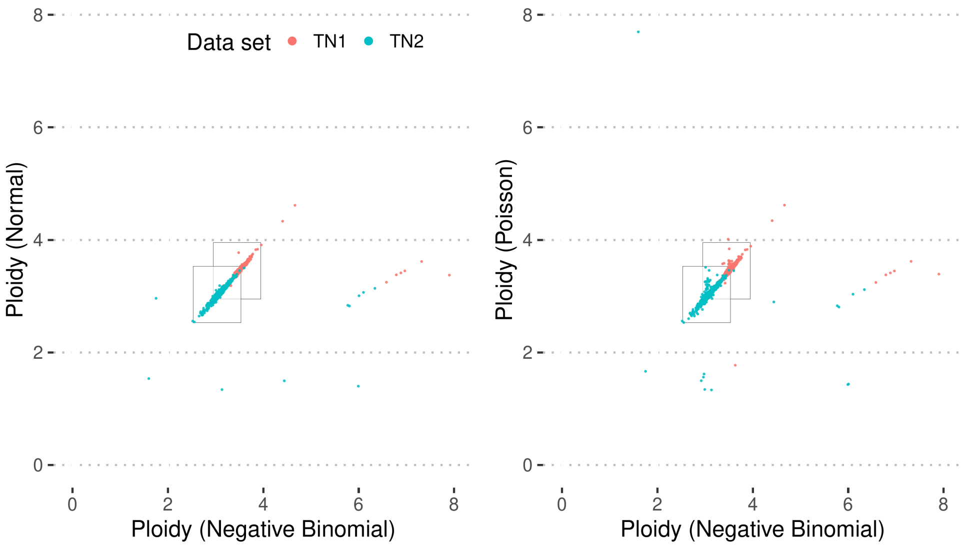

Fig. S16: **Impact of initial segmentation on ploidy calling.** We demonstrate that the algorithm is robust to the choice of initial segmentation algorithm in two example data sets (TN1 and TN2, bin size 500kb). The point estimate ($\pm$ 0.5) of FACS-based ploidy prediction is marked by black rectanges. Ploidy predictions mostly agree across the three different likelihoods (in parenthesis, Normal, Poisson, and Negative Binomial) chosen for the initial segmentation.
