# Supplementary material for: scAbsolute: measuring single-cell ploidy and replication status
Source: Genome Biol. 2024 Mar 4;25:62. doi: 10.1186/s13059-024-03204-y (PMC10910719; doi:10.1186/s13059-024-03204-y)
Supplement: Supplementary file 2 — Additional file 2. Supplementary methods. [file 13059_2024_3204_MOESM2_ESM.pdf]

Additional file 2: Supplementary methods

Derivation of Dirichlet Process Gaussian Mixture Model for scAbsolute algorithm

We implement a DPGMM model, following [68]. We assume throughout the document, that the dimensionality of our problem is 1. We use the mean-field approximation to estimate $p(\theta|X) = \prod_{k=1}^{K} \pi_k \, \mathcal{N}(\mu_k, \Lambda_k|X)$. The model is depicted below. Note that we restrict the means $\mu_k = \xi \cdot k$. K refers to the number of components obtained after fitting the model (T is the maximum possible number), and π_k refers to the normalized weight of the components.

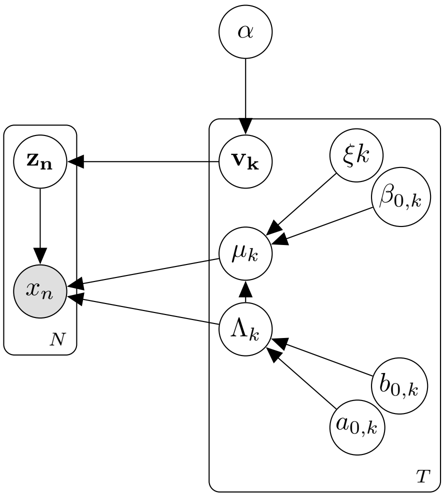

Model and priors

Mean-field approximation

$$Q(V, \mu, \Lambda, Z) = \prod_{k}^{T} q(v_k) q(\mu_k) q(\Lambda_k) \prod_{n=1}^{N} q(z_n) \tag{6}$$

Prior distributions.

$$v_k \sim \text{Beta}(1, \alpha) \tag{7}$$
$$\mu_k \sim \text{Normal}(m_{0,k}, \mathbf{I}) \tag{8}$$
$$\Lambda_k \sim \text{Gamma}(1, 1) \tag{9}$$
$$z_n \sim \text{SBP}(V) \tag{10}$$
$$x_n \sim \text{Normal}(\mu_{z_n}, \Lambda_{z_n}) \tag{11}$$

Variational distributions.

$$v_k \sim \text{Beta}(\gamma_{k,1}, \gamma_{k,2}) \tag{12}$$
$$\mu_k \sim \text{Normal}(\xi k, \mathbf{I}) \tag{13}$$
$$\Lambda_k \sim \text{Gamma}(a_k, b_k) \tag{14}$$
$$z_n \sim \text{Discrete}(\rho_n) \tag{15}$$

Variational bound

$$\log p(X) \geq \sum_{k=1}^{T} \mathbb{E}_q[\log p(v_k)] - \mathbb{E}_q[\log q(v_k)] \tag{16}$$
$$+ \sum_{k=1}^{T} \mathbb{E}_q[\log p(\mu_k) - \log q(\mu_k)] \tag{17}$$
$$+ \sum_{k=1}^{T} \mathbb{E}_q[\log p(\Lambda_k) - \log q(\Lambda_k)] \tag{18}$$
$$+ \sum_{n=1}^{N} \mathbb{E}_q[\log p(z_n|V) - \log q(z_n)] \tag{19}$$
$$+ \sum_{n=1}^{N} \mathbb{E}_q[\log p(x_n|\mu_{z_i}, \Lambda_{z_i})] \tag{20}$$

In the following, we derive the terms in the variational bound.

v_k *terms*

$$\mathbb{E}_q[\log p(V|1,\alpha)] = \mathbb{E}_q[\log \prod_{i=1}^{T} V_i] \tag{21}$$
$$= \mathbb{E}_q[\sum_{i=1}^{T} \ln V_i] = \tag{22}$$
$$= \mathbb{E}_q[\sum_{i=1}^{T} \ln \frac{\Gamma(1+\alpha)}{\Gamma(1)\Gamma(\alpha)} V_i^0 (1-V_i)^{(\alpha-1)}] = \tag{23}$$
$$= \mathbb{E}_q[\sum_{i=1}^{T} \ln \Gamma(1+\alpha) - \sum_{i=1}^{T} \ln \Gamma(\alpha) + \sum_{i=1}^{T} (\alpha-1)\ln(1-V_i)] \tag{24}$$
$$= \mathbb{E}_q[T(\ln \Gamma(1+\alpha) - \ln \Gamma(\alpha)) + (\alpha-1)\sum_{i=1}^{T} \ln(1-V_i)] \tag{25}$$

$$= T(\ln\Gamma(1+\alpha) - \ln\Gamma(\alpha)) + (\alpha-1)\sum_{i=1}^{T}\mathbb{E}_q[\ln(1-V_i)] \tag{26}$$

$$= T(\ln\Gamma(1+\alpha) - \ln\Gamma(\alpha)) \tag{27}$$

$$+ (\alpha-1)\sum_{i=1}^{T}[\Psi(\gamma_{i,2}) - \Psi(\gamma_{i,1}+\gamma_{i,2})] \tag{28}$$

$$\mathbb{E}_q[\log q(V|\gamma_1,\gamma_2)] = \mathbb{E}_q[\log\prod_{i=1}^{T} V_i] \tag{29}$$

$$= \mathbb{E}_q[\sum_{i=1}^{T}\ln V_i] \tag{30}$$

$$= \mathbb{E}_q[\sum_{i=1}^{T}\ln\frac{\Gamma(\gamma_1+\gamma_2)}{\Gamma(\gamma_1)\Gamma(\gamma_2)} V_i^{\gamma_1-1}(1-V_i)^{(\gamma_2-1)}] \tag{31}$$

$$= \mathbb{E}_q[\sum_{i=1}^{T}\ln\Gamma(\gamma_1+\gamma_2) - \ln\Gamma(\gamma_1) - \ln\Gamma(\gamma_2) \tag{32}$$

$$+ (\gamma_1-1)\ln(V_i) + (\gamma_2-1)\ln(1-V_i)] \tag{33}$$

$$= \sum_{i=1}^{T}\ln\Gamma(\gamma_1+\gamma_2) - \ln\Gamma(\gamma_1) - \ln\Gamma(\gamma_2) \tag{34}$$

$$+ (\gamma_1-1)\mathbb{E}_q[\ln(V_i)] + (\gamma_2-1)\mathbb{E}_q[\ln(1-V_i)] \tag{35}$$

$$= \sum_{i=1}^{T}\ln\Gamma(\gamma_1+\gamma_2) - \ln\Gamma(\gamma_1) - \ln\Gamma(\gamma_2) \tag{36}$$

$$+ (\gamma_1-1)(\Psi(\gamma_{i,1}) - \Psi(\gamma_{i,1}+\gamma_{i,2})) \tag{37}$$

$$+ (\gamma_2-1)(\Psi(\gamma_{i,2}) - \Psi(\gamma_{i,1}+\gamma_{i,2})) \tag{38}$$

$$\tag{39}$$

$$\sum_{k=1}^{T}\mathbb{E}_q[\log p(v_k)] - \mathbb{E}_q[\log q(v_k)] = T(\ln\Gamma(1+\alpha) - \ln\Gamma(\alpha)) \tag{40}$$

$$+ (\alpha-1)\sum_{k=1}^{T}[\Psi(\gamma_{k,2}) - \Psi(\gamma_{k,1}+\gamma_{k,2})] \tag{41}$$

$$- \sum_{k=1}^{T}\ln\Gamma(\gamma_{k,1}+\gamma_{k,2}) + \ln\Gamma(\gamma_{k,1}) + \ln\Gamma(\gamma_{k,2}) \tag{42}$$

$$-\sum_{k=1}^{T}(\gamma_{k,1}-1)(\Psi(\gamma_{k,1})-\Psi(\gamma_{k,1}+\gamma_{k,2})) \tag{43}$$

$$-\sum_{k=1}^{T}(\gamma_{k,2}-1)(\Psi(\gamma_{k,2})-\Psi(\gamma_{k,1}+\gamma_{k,2})) \tag{44}$$

μ_k ***terms***

$$\mathbb{E}_q[\log p(\mu_k)-\log q(\mu_k)] = -\mathrm{KL}(Q_{\mu_k}||P_{\mu_k}) \tag{45}$$

$$= -\frac{1}{2}||\xi k - m_{o,k}||^2 = -\frac{1}{2}(\xi k - m_{o,k})^2 \tag{46}$$

Λ_k ***terms***

We use the inverse scale parameter characterization of the Gamma distribution, with $\Lambda_k \sim \mathbb{G}(\Lambda_k|a_{k,0},b_{k,0})$, for $a_{k,0},b_{k,0}=1$, and $\mathbb{E}_q[\Lambda_k]=\frac{a_k}{b_k}$.

$$\mathbb{E}_q[\log p(\Lambda)] = \mathbb{E}_q[\log\prod_{k=1}^{T}\mathbb{G}(\Lambda_k|a_{k,0},b_{k,0}) \tag{47}$$

$$= \mathbb{E}_q[\sum_{k=1}^{T}\Big(a_{k,0}\log(b_{k,0}+(a_{k,0}-1)\log(\Lambda_k)-b_{k,0}\Lambda_k-\log(\Gamma(a_{k,0}))\Big)] \tag{48}$$

$$= \sum_{k=1}^{T}\Big(a_{k,0}\log(b_{k,0})+(a_{k,0}-1)\mathbb{E}_q\log(\Lambda_k)-b_{k,0}\mathbb{E}_q\Lambda_k-\log\Gamma(a_{k,0})\Big) \tag{49}$$

$$= \sum_{k=1}^{T}\Big(a_{k,0}\log(b_{k,0})+(a_{k,0}-1)(\Psi(a_k)-\log(b_k))-b_{k,0}\frac{a_k}{b_k}-\log(\Gamma(a_{k,0}))\Big) \tag{50}$$

$$\overset{a_{0,k}=b_{0,k}=1}{=} \sum_{k=1}^{T}\frac{a_k}{b_k} \tag{51}$$

$$\mathbb{E}_q[\log q(\Lambda_k)] \overset{\text{neg. Entropy}}{=} -a_k+\log b_k-\log\Gamma a_k-(1-a_k)\Psi(a_k) \tag{52}$$

$$\mathbb{E}_q[\log p(\Lambda_k)-\log q(\Lambda_k)] = a_k-\log b_k+\log\Gamma a_k+(1-a_k)\Psi(a_k)-\frac{a_k}{b_k} \tag{53}$$

z_n ***terms***

Here, we use the equations as presented by [68, p. 129].

$$\mathbb{E}_q[\log p(z_n|V)] = \sum_{k=1}^{T} f(z_n>k)\ \mathbb{E}_q[\log(1-v_k)] + f(z_n=k)\ \mathbb{E}_q[\log v_k] \tag{54}$$

$$= \sum_{k=1}^{T} \sum_{j=k+1}^{T} \phi_{n,j} \Big(\Psi(\gamma_{k,2}) - \Psi(\gamma_{k,1} + \gamma_{k,2}) \Big) + \tag{55}$$

$$\phi_{n,k} \Big(\Psi(\gamma_{k,1}) - \Psi(\gamma_{k,1} + \gamma_{k,2}) \Big) \tag{56}$$

with the following definitions

$$f(z_n = k) = \phi_{n,k} \tag{57}$$

$$f(z_n > k) = \sum_{j=k+1}^{T} \phi_{n,j} \tag{58}$$

$$\mathbb{E}_q[\log v_k] = \Psi(\gamma_{k,1}) - \Psi(\gamma_{k,1} + \gamma_{k,2}) \tag{59}$$

$$\mathbb{E}_q[\log(1 - v_k)] = \Psi(\gamma_{k,2}) - \Psi(\gamma_{k,1} + \gamma_{k,2}) \tag{60}$$

$$\mathbb{E}_q[\log q(z_n)] = \sum_{n=1}^{N} \phi_{n,k} \log(\phi_{n,k}) \tag{61}$$

$$\mathbb{E}_q[\log p(z_n) - \log q(z_n)] = \tag{62}$$

$$= \sum_{k=1}^{T} \Big(- \phi_{n,k} \log(\phi_{n,k}) \Big) \tag{63}$$

$$+ \sum_{k=1}^{T} \phi_{n,k} \Big(\Psi(\gamma_{k,1}) - \Psi(\gamma_{k,1} + \gamma_{k,2}) \Big) \tag{64}$$

$$+ \sum_{j=k+1}^{T} \phi_{n,j} \Big(\Psi(\gamma_{k,2}) - \Psi(\gamma_{k,1} + \gamma_{k,2}) \Big) \tag{65}$$

Derivation: (note this leads to a slightly different result than presented in Blei.)

$$P(z_n = k) = v_k \prod_{j=1}^{k-1} 1 - v_j \tag{66}$$

We use the following identities of the Beta distribution: for $B \sim \text{Beta}(b_1, b_2)$

$$\mathbb{E}[\log B] = \Psi(b_1) - \Psi(b_1 + b_2) \tag{67}$$

$$1 - B \sim \text{Beta}(b_2, b_1) \tag{68}$$

$$\mathbb{E}_q[\log(p(z_n|V) - \log(q(z_n)] = \mathbb{E}_z\Big[\mathbb{E}_v[\log V_{z_n} + \sum_{j=1}^{z_n-1}\log(1-v_j) - \log(\rho_n)]\Big] \tag{69}$$

$$= \mathbb{E}_z\Big[\mathbb{E}_v[\log V_{z_n}] + \sum_{j=1}^{z_n-1}\mathbb{E}_v[\log(1-v_j)] - \log(\rho_n)\Big] \tag{70}$$

$$= \mathbb{E}_z\Big[\Psi(\gamma_{z_n,1}) - \Psi(\gamma_{z_n,1}+\gamma_{z_n,2}) + \sum_{j=1}^{z_n-1}\mathbb{E}_v[\log(1-v_j)] - \log(\rho_n)\Big] \tag{71}$$

x_n *terms*

$$\mathbb{E}_q[\log p(x_n|\mu_{z_n}, \Lambda_{z_n})] \tag{72}$$

$$= \sum_{k=1}^{T}\phi_{n,k}\mathbb{E}_{\Lambda_k}[\mathbb{E}_{\mu_k}[\log P(x_n|\mu_k, \Lambda_k)] \tag{73}$$

$$= \sum_{k=1}^{T}\phi_{n,k}\mathbb{E}_{\Lambda_k}[\mathbb{E}_{\mu_k}[\log \mathbb{N}(x_n;\mu_k, \Lambda_k^{-1})] \tag{74}$$

$$= \sum_{k=1}^{T}\phi_{n,k}\mathbb{E}_{\Lambda_k}\left[\int_{\mu_k}\mathbb{N}(\mu_k;\xi,\mathbf{I})\log\mathbb{N}(\mathbf{x_n};\boldsymbol{\mu_k},\boldsymbol{\Lambda_k^{-1}})\mathbf{d}\boldsymbol{\mu_k}\right] \tag{75}$$

$$= \sum_{k=1}^{T}\phi_{n,k}\mathbb{E}_{\Lambda_k}\left[\int_{\mu_k}(2\pi)^{-\frac{1}{2}}\exp(-\frac{1}{2}(\mu_k - \xi k)^2)\right. \tag{76}$$

$$\left.\Big(-\frac{1}{2}\log(\frac{2\pi}{\Lambda_k}) - \frac{\Lambda_k}{2}(x_n-\mu_k)^2\Big)d\mu_k\right] \tag{77}$$

$$= \sum_{k=1}^{T}\phi_{n,k}\mathbb{E}_{\Lambda_k}\left[-\frac{1}{2}\log(2\pi) + \frac{1}{2}\log(\Lambda_k) + \right. \tag{78}$$

$$\left.\left(\frac{1}{2}\int_{\mu_k}(2\pi)^{-\frac{1}{2}}\exp(-\frac{1}{2}(\mu_k - \xi * k)^2)(-\Lambda_k(x_n^2 - 2\mu_k x_n + \mu_k^2)d\mu_k\right)\right] \tag{79}$$

$$= \sum_{k=1}^{T}\phi_{n,k}\mathbb{E}_{\Lambda_k}\left[-\frac{1}{2}\log(2\pi) + \frac{1}{2}\log(\Lambda_k) + \right. \tag{80}$$

$$\left. -\frac{1}{2}\Lambda_k((x_n - \xi k)^2 + 1)\right] \tag{81}$$

$$= \sum_{k=1}^{T}\phi_{n,k}\left[-\frac{1}{2}\log(2\pi) + \frac{1}{2}\Psi(a_k) - \frac{1}{2}\log(b_k) + \right. \tag{82}$$

$$-\frac{1}{2}\frac{a_k}{b_k}((x_n-\xi k)^2+1)\Bigg] \tag{83}$$

$$\tag{84}$$

We will refer to the following term later on

$$\eta_x = -\frac{1}{2}\log(2\pi)+\frac{1}{2}\Psi(a_k)-\frac{1}{2}\log(b_k)+ \tag{85}$$

$$-\frac{1}{2}\frac{a_k}{b_k}((x_n-\xi k)^2+1) \tag{86}$$

Variational updates

Updates for v_k

These update equations are given in [68, p. 129].

$$\gamma_{k,1} = 1+\sum_{n=1}^{N}\phi_{n,k} \tag{87}$$

$$\gamma_{k,2} = \alpha+\sum_{n=1}^{N}\sum_{j=k+1}^{T}\phi_{n,j} \tag{88}$$

Updates for ξ

$$\frac{\delta L}{\delta \xi} = \sum_{k=1}^{T} -k(\xi k - m_{0,k}) + \sum_{n=1}^{N}\sum_{k=1}^{T}\phi_{n,k}\frac{ka_k}{b_k}(x_n-\xi k) \tag{89}$$

$$= -\sum_{k=1}^{T}k^2\xi + \sum_{k=1}^{T}km_{0,k} + \sum_{n=1}^{N}\sum_{k=1}^{T}\phi_{n,k}\frac{ka_k}{b_k}x_n - \sum_{n=1}^{N}\sum_{k=1}^{T}\phi_{n,k}\frac{k^2a_k}{b_k}\xi \stackrel{!}{=} 0 \tag{90}$$

$$\Rightarrow \xi = \frac{\sum_{k=1}^{T}km_{0,k}+\sum_{n=1}^{N}\sum_{k=1}^{T}\phi_{n,k}\frac{ka_k}{b_k}x_n}{\sum_{k=1}^{T}k^2+\sum_{n=1}^{N}\sum_{k=1}^{T}\phi_{n,k}\frac{k^2a_k}{b_k}} \tag{91}$$

Updates for a_k *and* b_k

$$\frac{\delta L}{\delta a_k} = 1+\Psi(a_k)+(1-a_k)\Psi'(a_k)-\Psi(a_k) \tag{92}$$

$$-\frac{1}{b_k}+\frac{1}{2}sum_{n=1}^{N}\phi_{n,k}\Psi'(a_k)-\frac{1}{2}\sum_{n=1}^{N}\phi_{n,k}\frac{1}{b_k}((\xi k-x_n)^2+1) \tag{93}$$

$$= 1+\Psi'(a_k)(1-a_k+\frac{1}{2}\sum_{n=1}^{N}\phi_{n,k})-\frac{1}{b_k}(1+\frac{1}{2}\sum_{n=1}^{N}\phi_{n,k}((\xi k-x_n)+1) \tag{94}$$

$$\text{using the fact that } \Psi \text{ is a monotonous function} \tag{95}$$

$$\Rightarrow a_k = 1 + \frac{1}{2}\sum_{n=1}^{N}\phi_{n,k} \tag{96}$$

$$\Rightarrow b_k = 1 + \frac{1}{2}\sum_{n=1}^{N}\phi_{n,k}((\xi k - x_n)^2 + 1) \tag{97}$$

Computing the partial derivative $\frac{\delta L}{\delta b_k}$ and setting the values of a_k and b_k to the above results satisfies the condition.

$$\frac{\delta L}{\delta b_k} = -\frac{1}{b_k} + \frac{a_k}{b_k^2} - \frac{1}{2}\sum_{n=1}^{N}\phi_{n,k}\frac{1}{b_k} + \frac{a_k}{b_k^2}\sum_{n=1}^{N}((\xi k - x_n)^2 + 1) \stackrel{!}{=} 0 \tag{98}$$

Updates for ρ_n

Here, we need to take into account the constraint $\sum_{k=1}^{T}\phi_{n,k} = 1$ We do this by using Lagrange multipliers.

$$\mathcal{L}(\rho_n, \lambda) = \sum_{k=1}^{T}\big(- \phi_{n,k}\log(\phi_{n,k}) + \sum_{j=k+1}^{T}\phi_{n,j}(\Psi(\gamma_{k,2}) - \Psi(\gamma_{k,1} + \gamma_{k,2})) \tag{99}$$

$$+ \phi_{n,k}(\Psi(\gamma_{k,1}) - \Psi(\gamma_{k,1} + \gamma_{k,2})) + \phi_{n,k}\eta_x\big) - \lambda(\sum_{k=1}^{T}\phi_{n,k} - 1) \tag{100}$$

$$\frac{\delta\mathcal{L}}{\delta\phi_{n,k}} = -1 - \log(\phi_{n,k} + \sum_{j=k+1}^{T}\phi_{n,j}(\Psi(\gamma_{k,2}) - \Psi(\gamma_{k,1} + \gamma_{k,2})) \tag{101}$$

$$+ (\Psi(\gamma_{k,1}) - \Psi(\gamma_{k,1} + \gamma_{k,2})) + \eta_x - \lambda \tag{102}$$

$$\frac{\delta\mathcal{L}}{\delta\lambda} = 1 - \sum_{k=1}^{T}\phi_{n,k} \tag{103}$$

$$\Rightarrow \phi_{n,k} = \frac{\exp(\eta_{z_{n,k}} + \eta_{x_{n,k}} - 1)}{\sum_{k=1}^{T}\exp(\eta_{z_{n,k}} + \eta_{x_{n,k}} - 1)} \tag{104}$$
