# Supplementary material for: scAbsolute: measuring single-cell ploidy and replication status
Source: Genome Biol. 2024 Mar 4;25:62. doi: 10.1186/s13059-024-03204-y (PMC10910719; doi:10.1186/s13059-024-03204-y)
Supplement: Supplementary file 3 — Additional file 3. Review history. [file 13059_2024_3204_MOESM3_ESM.docx]

**1^st^ round**

**Reviewer 1**

In this paper, Schneider and co-authors propose a novel approach - scAbsolute - to measure single-cell ploidy and replication status. The method provides the advantage of being based on single-cell DNA sequencing information only, measures single-cell ploidy and replication status, including whole-genome duplications. Comparison with a good set of related methods is provided and critically discussed.

I find the approach interesting and well described, with a sound rationale behind it. The paper is very well written and entertaining, and the figures are informative and of good quality. The methods are appropriate to the aims of the study and well described; necessary controls are included. Sufficient details are provided to allow replication. The conclusions are adequately supported by the data.

I do see a major limitation in the scarce availability of currently present scDNA datasets with high coverage in the absence of whole genome amplification, for which all the steps of scAbsolute are applicable. Therefore, at the current moment, the method is likely to be suitable for specialized subgroup of users.

Nevertheless, overall, I believe that the work is worth publishing, as the paper addresses broad problem could be of broad interest in the field due to .

Some points to address below:

- Performance metrics are not clearly presented. For example, 10min per cell seems quite long for thousands of cells and multiple samples, and albeit the possibility for parallelization across cells is mentioned, no sufficient info is provided on how long it would take to process a sample. How scalable is the software?
- I am curious how would scAbsolute perform on a ground-truth synthetic data mimicking cell states and WGD.

Minor
- p9 refers too many times to the absence of whole genome amplification.

**Reviewer 2**

Schneider et al. propose scAbsolute to estimate single-cell ploidy and replication status. Accuracy is assessed and benchmarked using experimental cell multiplets. I have two main points.

1) How can copy number gains from G1/G2 phase be separated from hyperploidy cells (not necessarily whole-genome doubling events)? The authors gave an outline shown in Figure S2, but there are not empirical results associated with it. Also, with regard to "If the DNA is not amplified, each read can be assigned to one strand of DNA in a cell" from Figure S2's caption and its corresponding section in the main paper, why cannot a read be mapped to the same DNA region and thus overlap if they are from two replicated DNAs in G1/G2? Additionally, how can whole-genome doubling events be detected and differentiated from doubling the sequencing depth? Lastly, Figure 1, which serves as the overview of the method, does not mention anything on replication status. Is this due to the identifiability issue?
2) Can the cells be pooled together for more accurate estimation of the ploidy/replication status? This is especially important for cancer genomics, since cells from the same subclones share the same copy number aberrations. This would be a needed addition if this is to be published in Genome Biology.
3) The authors mentioned 500Kb bin resolution - how is this selected and how robust is the result with regard to the bin size? How is change-point detection and segmentation carried out from here and on (is it to combine consecutive bins)?

**Authors’ response**

Reviewer 1

I do see a major limitation in the scarce availability of currently present scDNA datasets with high coverage in the absence of whole genome amplification, for which all the steps of scAbsolute are applicable. Therefore, at the current moment, the method is likely to be suitable for specialized subgroup of users.

To realize the full benefit of all features in *scAbsolute* it is indeed best to use single-cell data generated without the use of whole genome amplification. However, *scAbsolute* can also be applied to data from workflows that use whole genome amplification.

As most of the recently published data come from the DLP+ workflow, which doesn’t use whole-genome amplification, we believe *scAbsolute* will be useful for a broad range of users. For example, the vast majority of single-cell DNA sequencing data (in terms of the absolute number of cells sequenced) has been created with the DLP/DLP+ technology in the labs of Sohrab Shah and Sam Aparicio:

● Laks, E et al. "Clonal decomposition and DNA replication states defined by scaled single-cell genome sequencing." Cell 179.5 (2019): 1207-1221.

● Funnell, T. et al. Single-cell genomic variation induced by mutational processes in cancer. Nature 612, 106–115 (2022). https://doi.org/10.1038/s41586-022-05249-0 ● Salehi, S., et al. Clonal fitness inferred from time-series modelling of single-cell cancer genomes. Nature 595, 585–590 (2021). https://doi.org/10.1038/s41586-021-03648-3 The coverage of the datasets in these studies often lies in the range of rho=75-100 at 500 kb resolution.

The other large source of published single-cell DNA sequencing data consists of ACT data, which are sequenced very shallowly and with single-end reads, but our approach is nevertheless, in theory, applicable to this technology, because it uses the same tagmentation-based, whole-genome amplification free approach that is similar to the DLP/DLP+ technology.

● Darlan C., et al. "Breast tumours maintain a reservoir of subclonal diversity during expansion." Nature 592.7853 (2021): 302-308

Among other technologies, the largest collection of data is available for the 10X technology. Step 4 of *scAbsolute’s* computational approach is not applicable to 10X data, but this technology is also no longer commercially available and thus not relevant going forward. Other approaches

like scKaryo-seq tend to be too shallow for our approach, but the available data generated with this technology so far is also limited and relatively small compared to the DLP data sets.

Some points to address below:

- Performance metrics are not clearly presented. For example, 10min per cell seems quite long for thousands of cells and multiple samples, and albeit the possibility for parallelization across cells is mentioned, no sufficient info is provided on how long it would take to process a sample. How scalable is the software?

Regarding the method's performance, we have included a paragraph with example run times for processing real-world samples to illustrate run time information better.

*Running the analysis on 64 CPU cores (Intel Xeon E5-2698v4 @2.20GHz, 1MB bin size), analyzing the TN1 sample (ACT technology, 1100 cells) takes about 2hours, processing a Breast tumour sample (BREAST-A, 2000 cells) takes 3-4 hours, and the MKN45 Gastric cancer cell line (5000 cells) takes about 14 hours. Due to the independent processing of individual cells, the runtime scales approximately linearly with the number of CPUs available, so using 128 CPU cores would approximately half the run time. Differences between individual samples are mainly due to the different number of solutions the algorithm has to iterate over.*

- I am curious how would scAbsolute perform on a ground-truth synthetic data mimicking cell states and WGD.

Regarding the performance on synthetic ground truth data, we have applied this to a simulation study with SCICoNE (Kuipers, Jack, et al. "Single-cell copy number calling and event history reconstruction." BioRxiv, doi: https://doi.org/10.1101/2020.04.28.065755) that allows us to simulate copy number profiles of both diploid and tetraploid cells synthetically. Unfortunately, we are not aware of any way to realistically simulate cell cycle states using this or other existing software. We have added a supplementary figure showing that *scAbsolute* can recover the results in almost all cases (about 2% error rate) in the four synthetic data sets, see Figure S12.

In our experience, however, most current methods to simulate copy number profiles tend to be unrealistic. We believe that creating more realistic copy number simulators is, therefore, an important future area of research. This is why we have put an emphasis on evaluating our method on real-world data sets.

Minor

- p9 refers too many times to the absence of whole genome amplification.

We have addressed this in the manuscript.

Reviewer 2

Schneider et al. propose scAbsolute to estimate single-cell ploidy and replication status. Accuracy is assessed and benchmarked using experimental cell multiplets. I have two main points.

1) How can copy number gains from G1/G2 phase be separated from hyperploidy cells (not necessarily whole-genome doubling events)? The authors gave an outline shown in Figure S2, but there are not empirical results associated with it.

The empirical results for using read density depicted as a schema in Figure S2 can be found in Figure 2. The measure of read density in Figure 2 corresponds to the notion of how many overlapping reads there are in a given genomic region, illustrated in Figure S2. We have added a reference in Figure 2.

Also, with regard to "If the DNA is not amplified, each read can be assigned to one strand of DNA in a cell" from Figure S2's caption and its corresponding section in the main paper, why cannot a read be mapped to the same DNA region and thus overlap if they are from two replicated DNAs in G1/G2?

Our method with step 4 (using overlapping reads) can only identify the “true” ploidy of a sample, e.g., it can assign a normal cell in G2 state a ploidy of 2*ground ploidy, and will assign a cell in G1 state the accurate ground ploidy. It will also correctly assign a cell that just underwent whole genome doubling (i.e. has not acquired any additional copy number changes after the WGD) a ploidy of 2*ground ploidy.

Without other experimental information, *scAbsolute* cannot distinguish between a G2 cell and a cell with WGD, unless one uses the information about the proportion of cells to be expected in G2 phase of the cell cycle at any given time point. We have clarified this further in the Discussion section, where we write:

*One limitation of the computational approach is the inability to distinguish cases of cells in G2 phase of the cell cycle from WGD cells. However, it is possible to address this either via recourse to estimates of the relative number of cells in G2 state, or via integration with experimental evidence, such as Geminin staining.*

Geminin staining differentially marks cells in G1 state compared to cells in G2/S phase of the cell cycle.

Additionally, how can whole-genome doubling events be detected and differentiated from doubling the sequencing depth?

Tagmentation divides the physical genome into separate reads. Note that this happens before genome amplification. In addition, we assume that the reads are approximately randomly distributed across the genome, i.e., the start positions are random. As a consequence, the number of overlapping reads will depend on the ploidy of the sample and the sequencing depth.

The approach to do this is depicted in Figure 2. The number of overlapping reads illustrated in Figure S2 is quantified by the read density measure.

By creating a model of expected read density given a sequencing depth (rho essentially captures the sequencing depth), we can predict what read density we would expect at a given sequencing depth. Deviations from this can only be explained by changes in ploidy.

Note that this is only possible for whole genome amplification-free protocols such as DLP or ACT, as mentioned in the manuscript.

Lastly, Figure 1, which serves as the overview of the method, does not mention anything on replication status. Is this due to the identifiability issue?

We do not include replication status in this figure, as replication status is estimated independently from the ploidy analysis. Replication status refers to cells in S phase of the cell cycle. The identifiability issue only affects cells in G1 vs G2, or cells who have undergone recent WGD.

2) Can the cells be pooled together for more accurate estimation of the ploidy/replication status? This is especially important for cancer genomics, since cells from the same subclones share the same copy number aberrations. This would be a needed addition if this is to be published in Genome Biology.

We do indeed pool the cells after the individual per-cell processing to determine per-cell replication status based on the distribution of the cycling activity measure (see Fig 3). We have made this point clearer in the method description.

*By using the distribution of the cells, we also obtain a sort of inherent regularization as we tend to observe a population of cells at different cell cycle stages.*

For ploidy estimation, we propose to estimate a per-cell ploidy initially. However, this forms part of an iterative approach where the output informs the selection of an accurate ploidy based on the predictions for all cells (see Figure 1). As a consequence, all the cells' ploidy informs the final ploidy choice for the cell population. We have also made this point clearer by explicitly adding it to the Figure description (Figure 1).

3) The authors mentioned 500Kb bin resolution - how is this selected and how robust is the result with regard to the bin size? How is change-point detection and segmentation carried out from here and on (is it to combine consecutive bins)?

500Kb is a common bin size in the literature (e.g. Funnell, Tyler, et al. "Single-cell genomic variation induced by mutational processes in cancer." Nature 612.7938 (2022): 106-115). Our method is generic with regard to the choice of bin size, as long as there are enough bins to run the Dirichlet Process Gaussian Mixture Model. In practice, this sets an upper limit of about 1000kb for the maximum bin size.

The optimal bin size is chosen such that sequencing depth per bin is large enough to easily detect changes in copy number status and such that there are enough bins to cluster the different copy number states effectively. We find 500kb to be a reasonable good default in practice; however, results are robust to a change in bin size (see Figure S13).

We have added the following to the manuscript to make this point clearer:

*Similarly, we found scAbsolute to be robust for different choices of bin sizes. Choice of bin size constitutes a trade-off between the accuracy of the segmentation and the number of bins. Generally, scAbsolute performs best for bin sizes of about 500kb and smaller, but can still be applied in cases with bin sizes of up to 1Mb, depending on how deeply the cells have been sequenced. Given sufficient read depth, we expect smaller bin sizes to produce better results, as the Gaussian approximation of copy number marginal distribution becomes better. However, very small bin sizes are often not practicable, because of costs associated with deeper sequencing (Fig. S13).*

Change-point detection and segmentation are not addressed in the manuscript. We focus on the issue of ploidy detection only.

Reviewer 3

With scAbsolute the authors present a novel tool and algorithm for estimating cellular ploidy from single-cell cancer sequencing data, with a particular emphasis on whole-genome amplification-free sequencing technologies, such as DLP.

With this article the authors address an important problem in a competitive field: Despite several available algorithms, the accurate detection of SCNAs and by extension of cellular ploidy remains challenging, the results noisy, and novel approaches are urgently needed.

We thank the reviewer for their valuable comments.

Despite this, I am having several issues with the paper in its current form that I believe need to be addressed. I will mostly comment on the computational and algorithmic aspects of the paper, since my expertise is in in this area.

1. Scope / concept: The authors mainly talk about ploidy estimation and I applaud the authors for clearly defining ploidy as the average CN state of the cell, not based on the number of chromosomes. However, if ploidy is the average CN along the genome of a cell, estimating ploidy is equivalent, or at least directly related to, estimating CN profiles (i.e. integer CN states for segments of the genome). However, due to the multiplicity issue, you need to know ploidy to estimate integer CN states, creating somewhat of a chicken and egg problem.

Now my issue with the paper how it is written is that I was never sure if scAbsolute is a CN caller (creating integer CN profiles) or "just" a ploidy estimator, or both. This leads to several points of confusion in the paper where as a reader I always wondered if ploidy is meant as a property of the cell or whether really CN state per bin / segment is of interest (e.g. Fig S2). Clearly, Step 1-3 of the algorithm are straightforward standard steps for inferring CN profiles (in bulk and single-cells), but with the caveat that apparently Step 1 is only a "coarse segmentation". However, in the Discussion the authors state that e.g. BAF values could be used to derive allele-specific CN states from the "high quality total CN predictions scAbsolute provides". So apparently scAbsolute does provide high-quality CN predictions, not just ploidy?

The authors need to more clearly define the scope of scAbsolute, a ploidy estimator and/or a CN caller and adjust the paper accordingly and throughout to avoid confusing the reader.

In its essence, *scAbsolute* is “just” a ploidy caller. However, as the reviewer rightly points out, there is an intricate link between ploidy and copy number calling. However, the dependency is such that accurate copy number calling relies on accurate ploidy estimation in the first place, whereas a rough estimate of ploidy (+- 0.5) is a necessary but not sufficient condition for highly accurate copy number calling. This has been demonstrated in *Mallory, Xian F., et al. "Assessing*

*the performance of methods for copy number aberration detection from single-cell DNA sequencing data." PLoS Computational Biology 16.7 (2020): e1008012.* In essence, some methods might perform well in detecting breakpoints, but this is of little use for downstream applications that rely on absolute copy number estimates when the overall estimation of the resulting copy number is off by a multiplicative factor/wrong scaling.

The estimation of this multiplicative factor/scaling is the main purpose of *scAbsolute*, in the sense that the overall ploidy is accurate under transformations of the (2n/2n+1) kind that makes ploidy calling difficult in practice. However, the purpose of scAbsolute is not to produce ploidy solutions accurate to 3 digits because this can only be achieved with very accurate segmentations. This is also an important aspect of research but is not addressed in this manuscript. We have purposefully chosen a ploidy window of +- 0.5 for the evaluation in order to be able to ignore differences in the quality of segmentation between different methods that are outside of the scope of this manuscript but are able to focus on differences in the quality of the ploidy estimation, with a particular emphasis on difficult cases, such as WGDs, and real-world performance. Evaluation of segmentation is also not possible in these cases, as we rely on the FACS estimates, which are themselves limited in ploidy resolution.

We have removed the misleading sentence in the discussion and have added further clarifications across the manuscript:

*To address these challenges, we introduce scAbsolute, a computational method specifically targeted at inferring individual cells’ ploidy and replication status based on shallow single-cell DNA sequencing data alone. Note that scAbsolute is not intended to provide highly accurate copy number calls, but the focus is very much on the problem of ploidy determination.*

*We demonstrate the feasibility of distinguishing cells in different, previously unidentifiable ploidy states, including cells directly after undergoing WGD, and introduce a novel, robust way of determining cell ploidy based on a Dirichlet Process Gaussian Mixture Model.*

*scAbsolute aims to find a transform based on cell ploidy to convert values from a scale of read counts per bin to a scale of absolute copy number.*

*The identification of per-cell ploidy and with it, per-cell read depth lies at the basis of many downstream applications, such as copy-number segmentation, estimation of allele and haplotype-specific copy number states, and the building of tumour phylogenies based on copy-number profiles. By solving the ploidy problem, further progress in developing more accurate and reliable CNA calling methods seems feasible. scAbsolute is complementary to existing approaches that use evolutionary phylogenies to infer copy number changes in the past by providing more accurate global copy number input predictions.*

We have also made clear throughout the manuscript that we consider ploidy as a cellular property, rather than a per-bin prediction.

2. This immediately leads to my second issue: initial segmentation. The authors state that the focus of the initial segmentation is "not on obtaining a highly accurate segmentation, but rather an initial segmentation that allows us to identify an accurate ploidy estimate". Clearly the accuracy of the segmentation is directly related to the accuracy of the ploidy estimate. a) Can the authors quantify this relationship and show how much would they gain from a more accurate segmentation?

b) I couldn't find where this initial segmentation is refined to create the "high quality total CN predictions scAbsolute provides" the authors are referring to in the discussion. This also links issue (2) into my issue (1) where I am again not sure if the focus of the algorithm should not be on CN profile inference.

*a)* As detailed above, the focus of the manuscript is not segmentation. We achieve this by focusing on a rough estimate of ploidy in the form of a ploidy window of +- 0.5 that is robust to differences in initial segmentations.

However, the method is robust to the choice of initial segmentation. We further support this claim by rerunning the analysis with an initial segmentation using a Normal and a Poisson likelihood instead of the Negative Binomial likelihood. While this leads to different segmentations, the results are fairly similar, see Figure S16.

We have chosen the ploidy window of +- 0.5 because of the limited resolution of experimental methods for ploidy determination. The best evidence available is not sufficient to evaluate the ploidy outside of this coarse ploidy window.

*b)* We have clarified this by removing the sentence in the discussion that is indeed misleading (see above). We wanted to put an emphasis on the quality of the overall ploidy calling, not the bin-level segmentation accuracy.

3. Novelty: Related to these points I am concerned about which parts of the algorithm are truly novel. From my understanding the innovative part seems to be Step 4 of the algorithm where the "read density" is used in genome amplification-free sequencing data to directly infer ploidy states (or determine an upper bound). This is indeed an excellent observation and could be a very powerful tool. Has this never been reported before?

I did not go back to the full DLP literature to see if the observation regarding the number of overlapping reads has previously been used or observed, but the authors should make clear whether this is truly novel or cite the relevant literature otherwise. All other aspects of the algorithm seem very straightforward and in line with many other algorithms describing the relationship of read counts to CN states (e.g. ASCAT, Sclust, etc.).

Having searched the literature in depth, we did not find a previous report of overlapping reads being used to improve ploidy prediction. We have added a sentence to make this clearer.

*To our knowledge, this is the first time the concept of read density has been used in the context of ploidy prediction and single-cell DNA sequencing.*

While the reviewer is correct that the analysis of read overlaps is a major contribution of the paper, is it not its only novelty. Step 3 is different from existing approaches in that it incorporates a robust Dirichlet Process Gaussian Mixture Model that appears generally more robust and outperforms existing methods on real-world data sets in practice, where we do not use step 4 of the algorithm (see Figure 4).

We have made this clearer in the introduction, where we write:

*We demonstrate the feasibility of distinguishing cells in different, previously unidentifiable ploidy states, including cells directly after undergoing WGD, and introduce a novel, robust way of determining cell ploidy based on a Dirichlet Process Gaussian Mixture Model.*

4. Model assumptions: The authors need to be more explicit about the assumptions of their model and discuss these. For example, there seems to be an implicit assumption that a) the scaling factor rho is constant per cell and uniform along the genome, which it probably is not; and

b) that there is a linear relationship between read counts and copy-number, whereas I would have intuitively expected some form of "compression" towards the higher CN spectrum. These assumptions (and any additional ones that there are) need to be clearly stated, they need to be validated, and ideally the effect of violations of these assumptions need to be quantified.

We explicitly state the assumptions of the model on page 26 (Ploidy estimation), where we have written:

*Here, we assume the errors are normally distributed….*

a) rho corresponds to an average read depth per cell, bin, and copy number. As a per-cell average, it is indeed a cell constant. However, there exists an additional variable eta, that impacts the read depth per bin, in the form of gc and mappability variation along the genome. We try to reduce the impact of this variation as far as possible, which is described in detail in the Methods section (Bin-level quality control).

However, since we are working with cell averages, we can ignore the factor eta for the purpose of estimating rho. For our purposes the variation in gc and mappability only adds to the variance of the estimator but does not impact the mean of the estimate.

Another way to think about the assumption of normal errors:

We assume that the estimate of the segmentation means is unbiased. In the case of the mean, the average count value of a segment is indeed an unbiased estimator of the mean. This does not mean the segmentation itself is always correct, but that it doesn’t systematically underestimate or overestimate the mean. So while the estimate would be noisier if one were to change the amount of gc and map variation, it would still be unbiased.

We have added a sentence when introducing rho to make clear we consider the variable to be a per-cell constant and we have added a sentence to clarify the meaning of the normal error assumption.

Again, on page 26 we write:

*Note that we assume that observed reads scale linearly with copy number state, e.g. we expect to observe on average twice as many reads for copy number state 2 than for copy number state 1, …*

b) Linearity is a fairly common assumption in copy number modeling that fits very well with our empirical observations and model (in fact, we are not aware of any work that does not make this assumption). For example, it is clearly visible in the marginal distributions of the copy numbers in Figure 1, top left panel.

The compression referred to is a property of the variance, i.e. higher copy number states have higher variance due to overdispersion. However, this is a property of the variance, not the mean, and is of little importance to the algorithm described here. It would manifest itself in larger standard deviations for higher copy number states, but we model standard deviations independently for each peak.

5. Algorithm Step 2: In the introduction the authors state that previous tools required "odd or intermediate copy number states in the data" to resolve abiguities in ploidy estimation and suggest that scAbsolute would overcome this drawback. However, in non-DLP data this seems to be also true for scAbsolute. At least I don't see how you would distinguish between a genome consisting only of 1/2 states and one consisting only of 2/4 states. Please clarify.

This is correct. We require non-whole genome amplified data (such as DLP, ACT) to apply step 4 of the algorithm and to distinguish between 1/2 and 2/4 states. We make clear that step 4 is only applicable for DLP/ACT data, or generally data not relying on whole genome amplification. However, when such data is available, *scAbsolute* is the first tool to distinguish these cases based on sequencing data alone.

6. Whole-genome doubling: The authors repeatedly refer to WGD as cells with high ploidy. These are two related but different concepts. High ploidy can also be achieved by a series of segmental or chromosomal gains, without ever suffering a doubling of the genome. This means that while most WGD cells show elevated ploidy, by far not all cells with elevated ploidy have undergone WGD. Dentro et al. in PCAWG have detailed nicely how ploidy alone is not sufficient to detect WGDs and how LOH events need to be taken into account on a cohort level. Kaufmann et al. 2022 have extended on this to show that event-based evolutionary models can directly distinguish between high ploidy due to WGD and high ploidy due to a series of independent gain events. The authors should cite the relevant literature, and make this distinction clear. From the paper it does not seem that the authors can claim that scAbsolute can detect WGD events, but that it instead can distinguish between high and low ploidy cells (if DLP-like data is available), the former of which might be due to WGD.

We agree with the reviewer that high-ploidy does not imply WGD. We focus on WGD cells as an example to highlight where the issue of identifiability is very evident and as one of the reasons for high ploidy. When we refer to WGD in our context, we refer to single cells that have recently undergone WGD, and are currently undistinguishable genomically from cells in G2 phase, i.e. they do not harbour any copy number changes that would allow existing algorithms to infer their correct ploidy.

The reviewer describes an approach based on evolutionary phylogeny to reconstruct the history of a tumour. However, this is not the point of the *scAbsolute* approach.

In fact, *scAbsolute* is complementary to such approaches by providing more accurate and reliable input for such methods such as MEDICC2, that rely on input in the form of scaled (absolute) copy number estimates.

We detail how it is possible to detect WGDs using *scAbsolute* and additional experimental information (Gemini staining) or population level frequency of G1/G2 in principle. Due to the lack of experimental data to support this, we simulate this kind of data with artificial cell duplets (see Figure 2). This application is particularly exciting in cases where it is not possible to have sufficient cell numbers for a FACS based analysis. Our approach is very much focused on making the inference of ploidy without recourse to an evolutionary model. This is interesting in cases where the copy number changes are relatively recent, and there has not been enough time to acquire copy number changes to allow for such a distinction.

To our knowledge, *scAbsolute* is the only tool currently able to identify a recent whole genome doubling (without any subsequent copy number changes) based solely on computational inference, so WGDs are a nice practical example illustrating the limitations of existing methods in determining accurate copy number status.

We have added a paragraph to clarify this aspect on page 31.

Minor points:

1. Naming: scAbsolute immediately invokes associations to the similarly named "Absolute" algorithm for CN inference from bulk sequencing. Not sure this is intentional, since I don't see an overlap in authors or a similarity in the methodological approach.

Our method estimates absolute cell ploidy in single-cell data, which makes “scAbsolute” an intuitive name. There is indeed no link to the historic “Absolute” method for bulk data.

2. line 10: MEDICC2 (Kaufmann et al. Genome Biology 2022) should be cited. Thank you for noticing. We have updated the reference.

3. Fig 1: I would avoid fuzzy terms such as "hypotriploid" or "hypohexaploid" and instead simply use the cell-level ploidy value.

As pointed out in 1), we chose the names to refer to the ploidy window rather than an exact ploidy solution.

**2^nd^ round**

**Reviewer 2**

The authors have addressed my previous concerns .
